# Serum Midkine as a Biomarker for Hepatocellular Carcinoma Treatment Response and Prognostication

**DOI:** 10.1002/jcla.70255

**Published:** 2026-05-25

**Authors:** Yanfang Luo, Qiuwei Lu, Jing Huang, Liejun Jiang

**Affiliations:** ^1^ Department of Laboratory Medicine The People's Hospital of Guangxi Zhuang Autonomous Region Nanning Guangxi China; ^2^ Department of Laboratory Medicine The Third People's Hospital of Nanning Nanning Guangxi China

**Keywords:** alpha‐fetal protein (AFP), hepatocellular carcinoma (HCC), Midkine, prognostication, treatment response evaluation

## Abstract

**Background:**

Up‐to‐date tumor markers are considered more helpful in the evaluation of treatment response and prognostication than in diagnosis. Identification of a valid biomarker to help clinical management of hepatocellular carcinoma (HCC) is still a challenge, especially in alpha‐fetoprotein (AFP) negative HCC. This study evaluated the efficacy of serum Midkine in HCC treatment response and prognostication as well as its association with clinical pathological features.

**Methods:**

The enzyme‐linked immunosorbent assay (ELISA) was used to analyze the serum (soluble) Midkine levels in patients with benign and malignant liver disease and healthy subjects.

**Results:**

Serum Midkine levels in HCC, hepatic cholangiocarcinoma (HC), chronic hepatitis B (CHB), and decompensated hepatic cirrhosis (DHC) patients were significantly higher than in healthy subjects and patients with benign hepatic tumors, with HCC being the highest. Serum Midkine levels were associated with patients' clinical pathologic features such as tumor size, ascites, high Child‐Pugh and BCLC stages, and advanced clinical stages. Serum Midkine was helpful in characterizing the AFP negative HCC patients. A model has been created using univariate and multivariate regression analysis of biomarkers in predicting HCC, which showed that Midkine was one of the key predictive factors in AFP negative HCC. Midkine levels as a parameter for treatment response evaluation and disease progression/prognostication are comparable to computed tomography scan (CT) and magnetic resonance imaging (MRI), especially in non‐surgical treatment patients.

**Conclusion:**

Serum Midkine levels could be used as a parameter for evaluation of treatment efficacy and prognostication of HCC, especially in AFP‐negative HCC.

## Graphical abstract

Up‐to‐date, tumor markers are considered more helpful in the evaluation of treatment response and prognostication rather than diagnosis. Identifying a valid biomarker to help clinical management of hepatocellular carcinoma (HCC) is still a challenge, especially the alpha‐fetoprotein (AFP) negative HCC. This study evaluated the efficacy of serum Midkine in HCC treatment response and prognostication. The enzyme‐linked immunosorbent assay (ELISA) was used to analyze the serum (soluble) Midkine levels in patients with benign and malignant liver disease and healthy subjects. Serum Midkine levels in HCC, hepatic cholangiocarcinoma (HC), chronic hepatitis B (CHB), and decompensated hepatic cirrhosis (DHC) patients were significantly higher than in healthy subjects and patients with benign hepatic tumors, with HCC being the highest. Serum Midkine levels were associated with patients' clinical pathological features such as tumor size, ascites, high Child‐Pugh and BCLC stages, and advanced clinical stages. Serum Midkine was helpful in characterizing the AFP negative HCC patients. Univariate and multivariate regression analysis showed that Midkine was a key predictive factor in AFP negative HCC prediction. Midkine level as a parameter for treatment response and disease progression/prognostication evaluation in comparison with computed tomography (CT) scan and magnetic resonance imaging (MRI), especially in non‐surgical resection treatment patients. Serum Midkine and AFP levels could be used as a parameter for evaluation of treatment efficacy and prognostication of HCC, especially in AFP negative HCC.

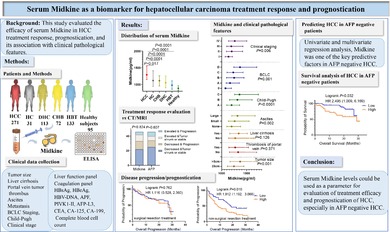

## 1 Introduction

Liver cancer, especially hepatocellular carcinoma (HCC), is still a leading cause of death among all types of cancers worldwide, and China is one of the most affected countries for HCC [1, 2, 3]. With advances in diagnosis and treatment, the 5‐year survival rate of liver cancer is still very low [4, 5], so a valid biomarker to help clinical management of HCC is still key in increasing the survival rate [6, 7]. Traditional monitoring such as ultrasound is a common tool; however, the detection efficacy of ultrasound relies heavily on the operator's experience [8, 9]. Other imaging techniques such as magnetic resonance imaging (MRI) and computed tomography scan (CT) are too costly to access and lack sensitivity and specificity [10, 11]. Serum biomarker alpha‐fetoprotein (AFP) is commonly used in liver cancer diagnosis and management; however, the sensitivity of AFP is about 65%, and 30%–40% of HCC are AFP negative [12, 13, 14]. Other serum biomarkers, such as the AFP isoform *Lens culinaris* agglutinin‐reactive fraction of AFP (AFP‐L3) and prothrombin induced by vitamin K deficiency or antagonist‐ II (PIVKA‐II, also called des‐gamma‐carboxy prothrombin (DCP)), have been developed and used in combination with AFP to enhance diagnostic power and management monitoring; however, various challenges remain when using these parameters in clinical liver cancer management [15, 16, 17]. Thus, the finding of new liver cancer biomarkers is still a challenge and priority.

Midkine is a heparin‐binding growth factor which is considered a pan‐tumor and inflammatory multifaceted marker. Some authors suggest that it is a marker of both normal tissue homeostasis and disease development [18, 19]. Studies have reported that Midkine was elevated either in tissue or in serum in multiple malignancies including breast, ovarian, uterine/cervical, prostate, lung, neuroblastoma, glioblastoma, meningioma, neurofibromatosis type 1, gastric, GI stromal, bladder, colorectal, duodenal, oral squamous cell, esophageal squamous cell, bile duct, pancreatic, renal, thyroid, and chronic lymphocytic leukemia [20, 21, 22, 23, 24, 25, 26, 27, 28]. In hepatocellular carcinoma, serum Midkine levels were upregulated in hepatocellular carcinoma patients in multiple studies [28, 29, 30, 31, 32, 33, 34, 35, 36, 37, 38, 39, 40, 41, 42, 43, 44, 45, 46, 47, 48, 49]. However, all of the above‐mentioned studies either lacked a comprehensive analysis of the association/relationship between Midkine and clinical pathological features or lacked a comparison with benign‐disease groups and other biomarkers for liver cancer; previous studies also had small sample sizes with heterogeneous reported sensitivity, specificity, and cut‐off values. Also, previous studies focused on diagnosis rather than treatment response evaluation and prognostication. Herein, we report a comprehensive evaluation of serum Midkine as a potential biomarker for use in liver cancer, especially in HCC treatment response, disease severity evaluation, and prognostication. Its association with clinical pathological features was also presented.

## 2 Patients and Methods

### 2.1 Project Design and Ethical Approval

This study enrolled 271 hepatocellular carcinoma (HCC), 72 chronic hepatitis B (CHB), 113 decompensated hepatic cirrhosis (DHC), 132 hepatic benign tumors (HBT), and 31 hepatic cholangiocarcinoma (HC) patients. All were admitted for treatment at the People's Hospital of Guangxi Zhuang Autonomous Region from April 2023 to October 2024.

This study adhered to the Declaration of Helsinki for study involving humans or human data. The Ethic Committee/Institutional Review Board of the People's Hospital of Guangxi Zhuang Autonomous Region approved this study (No. KY‐SY‐2002‐007). Patients' informed consent was waived because the study only used patients' serum left over from routine testing, and patients' identities were not disclosed. For HCC patients, 234 (86%) were male with age ranging from 28 to 87 years, and 37 were female, with age ranging from 26 to 88 years. For CHB patients, 38 (53%) were male with age ranging from 23 to 76 years, and 34 were female with age ranging from 6 to 68 years. For DHC patients, 82 (73%) were male with age ranging from 29 to 77 years, and 31 were female with age ranging from 34 to 76 years. For HBT patients, 64 (48%) were male with age ranging from 21 to 86 years, and 68 were female with age ranging from 26 to 88 years. For HC patients, 16 (52%) were male with age ranging from 40 to 82 years, and 15 were female with age ranging from 37 to 82 years.

Inclusion and exclusion criteria: The diagnosis of HCC followed the Guidelines of Chinese expert consensus on refined diagnosis, treatment, and management of advanced primary liver cancer (2023 edition) [50]. All patients were enrolled before surgical resection, chemotherapy, trans arterial chemoembolization (TACE), immuno‐therapy, targeted cancer drugs, radiotherapy, and integrative Chinese and Western medicine (ICWM) treatments. All patients were negative for human immunodeficiency virus (HIV) and Ebstein‐Barr virus (EB), and had no other malignancies, chronic kidney disease, severe cardiovascular diseases, rheumatoid arthritis, or auto‐immune diseases.

Patients grouping: HCC patients group included both hepatitis B virus positive and negative status. Patients with liver cirrhosis, chronic hepatitis, and liver hemangiomas were enrolled as comparisons. The diagnosis of liver cirrhosis was based on blood chemistry, ultrasound, and MRI, and the severity evaluation was defined by Child Pugh scores. The chronic hepatitis B infection group did not include patients with liver cirrhosis. Healthy control group consisted of normal subjects with age spans matching the disease groups and were HBsAg negative.

### 2.2 Clinical Data Collection

Tumor size, evidence of liver cirrhosis, portal vein tumor thrombus, metastasis (including lymph node and distant organ metastasis), the Barcelona Clinic Liver Cancer (BCLC) Staging, Child‐Pugh staging, liver function panel, coagulation panel, HBsAg, HBeAg, APF, PIVK1‐II, AFP‐L3, carcinoembryonic antigen (CEA), cancer antigen 125, cancer antigen 199, hepatitis B virus DNA, and complete blood count were collected.

### 2.3 Blood Sample Collection

Fasting blood sample was collected on the day following admission. Samples were delivered to the clinical laboratory for analysis within 2 h of collection.

### 2.4 Enzyme‐Linked Immunosorbent Assay (ELISA) of Midkine

ELISA was used for Midkine analysis (biotechne/Novus Biologicals, Minneapolis, MN, USA), and the assay kits were kindly provided by Shenzhen Mindray Bio‐Medical Electronics Co. Ltd. (Shenzhen, Guangdong, China). Briefly, 100 μL of each dilution of standard, blank, and sample was added to the appropriate wells of the plate. The plate was covered with the sealer and incubated for 90 min at 37°C. After the incubation, the liquid in each well was decanted, and 100 μL of biotinylated detection solution was added into each well. The wells were covered with a new sealer and incubated for 1 h at 37°C. The liquid in each well was decanted, and 350 μL of wash buffer was added and followed by soaking for 1 min, then the solution in all wells were aspirated or decanted and patted against clean absorbent paper to dry any remaining liquid. This wash was repeated three times. After washings, 100 μL of HRP conjugate working solution was added to each well and covered with a new sealer. The plate was then incubated for 30 min at 37°C. After this incubation, the solution in each well was decanted, and this washing was repeated five times. After the last washing, 100 μL of substrate mixture solution was added to each well. The plate was covered with a new plate sealer to protect it from light and incubated for no more than 5 min at 37°C. Lastly, the relative light unit (RLU) value was measured by the chemiluminescence immunoassay analyzer at 425 nm.

### 2.5 Hematology Analysis

Complete blood count was performed on a Sysmex XN9000 hematology analysis system (Sysmex, Kobe, Japan) following the manufacturer's instructions and laboratory standard operating procedures.

### 2.6 Clinical Chemistry Analysis

AFU, ALT, AST, γ‐GT, TBIL, DBIL, IBIL, total protein (TP), and ALB were analyzed on the AU5800 chemistry analyzer system (Beckman‐Coulter, Brea, CA, USA) following the manufacturer's instructions and clinical laboratory standard operating procedures.

### 2.7 Immunoassay of Hepatitis B and Tumor Markers

Hepatitis B surface antigen (HBsAg), AFP and its sub‐type, the AFP‐L3, CEA, CA‐199, CA‐724, and PIVKA‐II were analyzed on the Roche Cobas 8000 chemiluminescence immunoassay system (Roche, Indianapolis, IN, USA) and Architect i2000sr (Abbott, Lake Forest, IL, USA) following the manufacturer's instructions and clinical laboratory standard operating procedures.

### 2.8 Blood Coagulation Test

The prothrombin time (PT) test was performed on a STAGO‐R Evolution coagulation analyzer (Diagnostica STAGO S.A.S, Asnières sur Seine Cedex, France) following the manufacturer's instructions and clinical laboratory standard operating procedures.

### 2.9 Real Time Quantitative Polymer Chain Reaction (qPCR) Detection of Hepatitis B Virus DNA

Hepatitis B virus DNA was detected by real‐time qPCR on a Thermo Fisher Scientific ABI 7500 DNA Thermo Amplifier (Thermo Fisher Scientific, Waltham, MA, USA). Results were represented in copy number of the DNA as reported by the analyzer.

### 2.10 Pathological Diagnosis

The need for a pathological diagnosis of liver cancer depended upon discrepancies between oncologists and surgeons; thus, not all cases were pathologically diagnosed. Surgical tissues were sent to pathology for diagnosis when needed. The processes of pathological diagnosis were conducted following the standard operating procedures in routine clinical practice in pathology.

### 2.11 Treatment Efficacy Evaluation

A total of 46 consecutive hepatocellular carcinoma (HCC) cases (including both AFP‐negative and AFP‐positive HCC) were assessed for treatment efficacy 3 months after intervention. Routine liver CT/MRI examinations were performed to compare changes in tumor size before and after treatment, and patients were divided into two groups based on the presence of lymph node metastasis or venous tumor thrombus: (1) Tumor regression or stability; (2) Tumor progression (including tumor enlargement, emergence of new lesions, lymph node metastasis, or venous tumor thrombus formation). To ensure that the changes reflect true biological fluctuations rather than analytical bias, we set a 15% change threshold for AFP and Midkine before and after treatment based on several published studies [51, 52] and clinical practice.

### 2.12 Patient Survival Analysis

#### 2.12.1 Midkine Levels to Predict the Overall Survival for AFP Negative HCC Patients

Eighty‐four AFP negative HCC patients from April 2023 to May 2024 were included in the analysis. Follow‐up was conducted from diagnosis to January 2026. Midkine levels were grouped into low and high value groups based on Midkine median value (1291 pg/mL) in HCC patients, and Kaplan–Meier was used for the survival analysis.

#### 2.12.2 Midkine Levels to Predict HCC Progression After Treatment

Seventy‐six surgical resections and 126 non‐surgical treatments (Trans arterial Chemoembolization, hepatic microwave ablation, immune targeted therapy, and the combination therapy of the three) HCC patients from April 2023 to May 2024 were included in the analysis. Follow‐up was conducted from diagnosis to January 2026. Tumor progression was evaluated by magnetic resonance imaging or computed tomography scan. Midkine levels were grouped into low and high value groups based on Midkine median value (1291 pg/mL) in HCC patients, and Kaplan–Meier was used for the survival analysis.

### 2.13 Statistical Analysis

Statistical analysis was performed using SPSS Version 26 for Windows; the statistical analysis was conducted from October 2024 to December 2024. Specifically, statistical analysis was as follows: SPSS 26.0 and Graphic Prism 10 statistical software were used for data analysis. A *p* value < 0.05 was considered statistically significant. Mann–Whitney *U* test, Kruskal–Wallis Test, Pearson analysis, Binary logistic, Kaplan–Meier, and ROC (receiver operating characteristic curve) were used in the data analysis. A two‐tail analysis was adopted, and a *p* value < 0.05 was considered significant in difference.

## 3 Results

### 3.1 Basic Characteristics of Patient Groups

Table 1 shows a list of the basic characteristics of patient groups.

**TABLE 1:** Basic characteristics of patient groups.

		*n*	Median age	Age range
HCC	M	234	58	28–87
	F	37	60	26–88
	Total	271		
CHB	M	38	44	23–76
	F	34	42.5	6–68
	Total	72		
DHC	M	82	54.5	29–77
	F	31	56	34–76
	Total	113		
HBT	M	64	54.0	21–86
	F	68	51.5	26–88
	Total	132		
HC	M	16	65	40–82
	F	15	65	37–82

Abbreviations: CHB, chronic hepatitis B; DHC, decompensated hepatic cirrhosis; HBT, hepatic hemangiomatosis; HC, hepatic cholangiocarcinoma; HCC, hepatocellular carcinoma.

### 3.2 Serum Midkine Levels in Patient Groups and Healthy Controls

**FIGURE 1:**
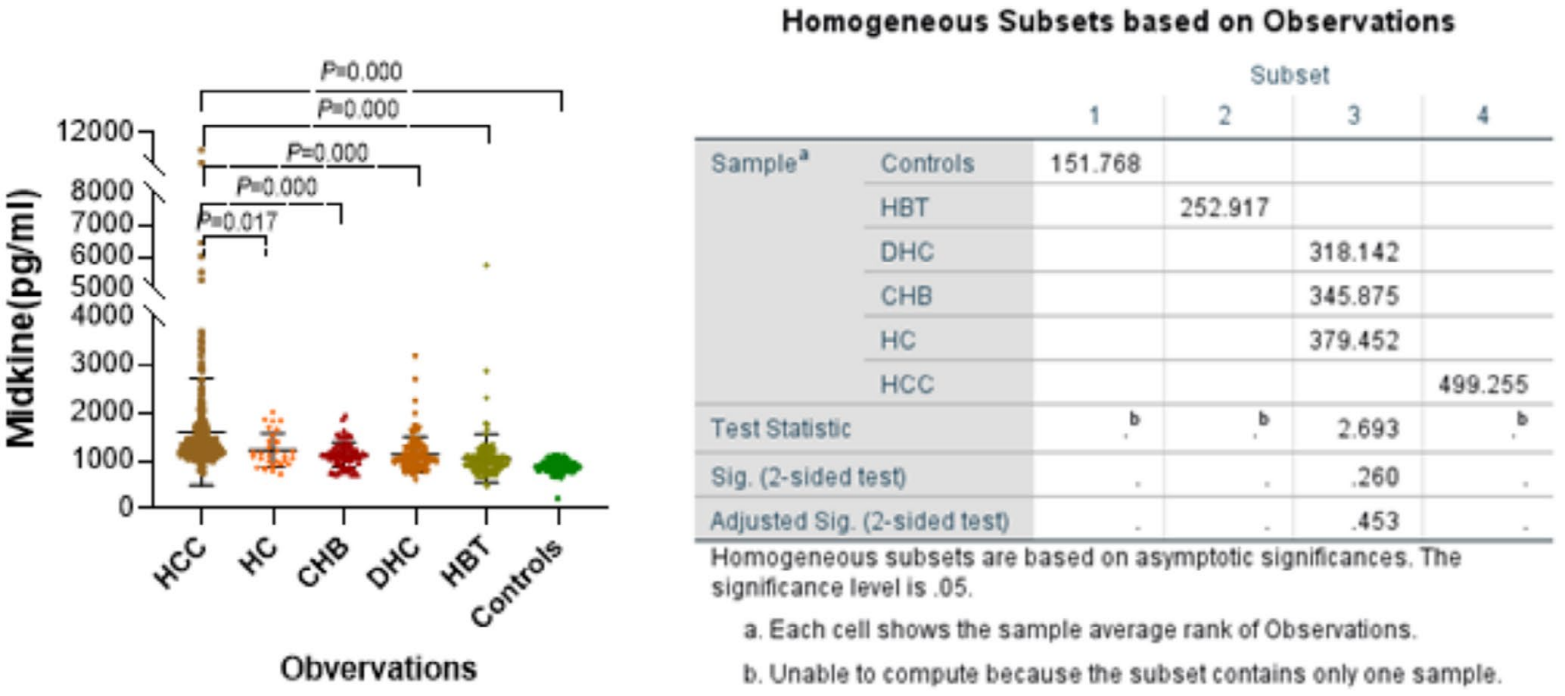
Serum Midkine levels among patient groups and controls (Kruskal–Wallis test).

Table 2 and Figure 1 show serum Midkine levels in patient groups and healthy controls. HCC patients had the highest Midkine levels, followed by cholangiocarcinoma, chronic hepatitis, liver cirrhosis, and benign liver tumor patients. Midkine levels were lowest in healthy controls.

**TABLE 2:** Serum Midkine in patient groups and healthy controls.

Groups	*n*	Median (pg/mL)	P25–P75 (pg/mL)	Mean rank	Test statistic	*p*
Healthy subjects	95	885	821–962	151.863	260.677	0.000
HCC	271	1291	1149–1554	499.253		
CHB	72	1114	956–1260	345.889		
DHC	113	1054	902–1257	318.155		
HBT	133	979	859–1112	255.937		

### 3.3 Association Between Serum Midkine Levels and Clinical Pathological Features

Table 3 shows the association between Midkine levels and clinical pathological features. Results showed that tumor sizes larger than 5 cm in diameter had higher serum Midkine levels than those with smaller than 5 cm diameter (*p* = 0.001); patients with ascites had higher serum Midkine levels than those without ascites (*p* = 0.002); patients with high Child‐Pugh scores, high BCLC stage, and advanced clinical stages had higher serum Midkine than those with low Child‐Pugh scores (*p* = 0.000), low BCLC stage (*p* = 0.001), and low clinical stages (*p* = 0.006). There was no significant difference in serum Midkine levels between patients with and without liver cirrhosis and between patients with and without portal vein thrombosis (*p* = 0.126 and 0.371, respectively).

**TABLE 3:** Association between serum Midkine levels and clinical pathological features.

Pathological features	Parameters	*n*	Median	P25–P75	Mean rank	Test statistic	*p*
Tumor size^a^ (largest in diameter)	≤ 5 cm	91	1244	1113–1444	108.15	−3.265	0.001
	> 5 cm	165	1368	1165–1709	139.72		
Liver cirrhosis^a^	No	91	1328	1152–1699	144.60	−1.53	0.126
	Yes	177	1269	1142–1535	129.31		
PVT^a^	No	181	1269	1269–1648	131.09	−0.894	0.371
	Yes	86	1340	1340–1512	140.13		
Ascites^a^	None	144	1246	1126–1473	120.12	12.179	0.002
	Small amount	69	1324	1165–1685	141.36		
	Large amount	54	1394	1251–1835	161.60		
Child‐Pugh^b^	A	160	1245	1127–1446	115.89	16.957	0.000
	B	77	1391	1195–1761	149.25		
	C	23	1444	1324–1915	169.33		
BCLC^b^	0	11	1113	1094–1291	67.18	18.866	0.001
	A	77	1247	1132–1525	116.66		
	B	29	1370	1144–1664	136.85		
	C	131	1343	1159–1633	137.21		
	D	12	1544	1360–2040	188.79		
Clinical stage^b^	I	48	1201	1105–1324	101.68	12.464	0.006
	II	57	1297	1142–1621	131.82		
	III	99	1286	1149–1630	135.61		
	IV	59	1389	1203–1746	153.48		

Abbreviations: BCLC, Barcelona clinic liver cancer staging; PVT, portal vein thrombosis.

^a^ Mann–Whitney *U*.

^b^ Kruskai–Wallis *H*.

### 3.4 Univariate and Multivariate Regression Analysis of the Value of Biomarkers in Predicting HCC in AFP Negative HCC (Binary Logistic Analysis)

To evaluate the value of markers in predicting HCC in AFP negative patients, univariate and multivariate regression analysis were performed, and the results of univariate regression analysis indicated that 20 out of 62 parameters (Table S1) were significant (Table 4), which were then further analyzed using multivariate regression analysis. Subsequently, 8 parameters were found to be significant (Table 5); the model formula was: −9.179 + 1.418 × Sex (Male) + 0.065 × Age + 0.002 × Midkine + 0.002 × PIVKA‐II + 2.670 × HBsAg (+) + 0.577 × RBC − 0.093 × MON% + 0.303 × GGT/ALP. The formula also indicated that HBsAg (+) was the highest contributor in HCC prediction.

**TABLE 4:** Univariate regression analysis to find HCC prediction factors in AFP negative HCC.

Factors	*B*	SE	Wald	*p*	OR (95% CI)
Sex	1.235	0.307	16.144	0.000	3.439 (1.883–6.281)
Age	0.059	0.010	37.814	0.000	1.060 (1.041–1.080)
Midkine	0.002	0.000	33.480	0.000	1.002 (1.001–1.002)
PIVKA‐II	0.003	0.001	16.258	0.000	1.003 (1.002–1.005)
AFP‐L3%	0.095	0.036	7.051	0.008	1.100 (1.025–1.179)
RBC	−0.260	0.131	3.939	0.047	0.771 (0.596–0.997)
HGB	−0.011	0.004	5.938	0.015	0.990 (0.981–0.998)
HCT	−0.040	0.015	6.746	0.009	0.961 (0.933–0.990)
PLT	−0.003	0.001	5.538	0.019	0.997 (0.995–1.000)
Lym%	−0.029	0.011	7.518	0.006	0.971 (0.951–0.992)
Mon%	0.041	0.018	5.115	0.024	1.042 (1.005–1.080)
Lym	−0.522	0.155	11.285	0.001	0.594 (0.438–0.805)
PDW	−0.114	0.045	6.404	0.011	0.893 (0.817–0.975)
AST	0.003	0.001	6.221	0.013	1.003 (1.001–1.005)
TP	−0.042	0.014	8.410	0.004	0.959 (0.933–0.987)
ALB	−0.087	0.017	25.865	0.000	0.916 (0.916–0.948)
A/G	−1.376	0.330	17.383	0.000	0.252 (0.132–0.482)
CHE	0.000	0.000	11.470	0.001	1.000 (1.000–1.000)
ADA	0.058	0.013	18.402	0.000	1.059 (1.032–1.088)
GGT/ALP	0.165	0.075	4.889	0.027	1.179 (1.019–1.365)

Abbreviations: A/G, serum albumin/globulin ratio; ADA, adenosine deaminase; ALB, serum albumin; ALT, alanine aminotransferase; AST, Aspartate aminotransferase; CHE, cholinesterase; GGT/ALP, gamma‐glutamyl transferase to alkaline phosphatase ratio; HCT, hematocrit; HGB, hemoglobin; Lym%, lymphocyte percentage; Lym#, lymphocyte count; Mon%, monocyte percentage; Mon#, monocyte count; OR, odds ratio; PDW, platelet distribution width; PLT, platelets; RBC, red blood cell; SE, standard error; TP, serum total protein.

**TABLE 5:** Multivariate regression analysis to find risk factors for HCC and establish a diagnostic model in AFP negative HCC.

Factors	*B*	SE	Wald	*p*	OR (95% CI)
Male sex	1.481	0.485	9.331	0.002	4.397 (1.700–11.371)
Age	0.065	0.018	13.114	0.000	1.067 (1.030–1.105)
Midkine	0.002	0.001	10.232	0.001	1.002 (1.001–1.003)
PIVKA‐II	0.002	0.001	6.775	0.009	1.002 (1.001–1.004)
HBsAg (+)	2.670	0.472	32.008	0.000	14.433 (5.724–36.391)
RBC	0.577	0.242	5.682	0.017	1.781 (1.108–2.863)
MON%	−0.093	0.045	4.221	0.040	0.911 (0.834–0.996)
GGT/ALP	0.303	0.114	7.025	0.008	1.355 (1.082–1.695)
Constant	−9.179	1.999	21.081	0.000	0.000

*Note:* Model formula: −9.179 + 1.418 × Gender (Male) + 0.065 × Age + 0.002 × Midkine + 0.002 × PIVKA‐II + 2.670 × HBsAg (+) + 0.577 × RBC − 0.093 × MON% + 0.303 × GGT/ALP.

### 3.5 Efficacy of Midkine Levels in Predicting the Overall Survival for AFP Negative HCC Patients

**FIGURE 2:**
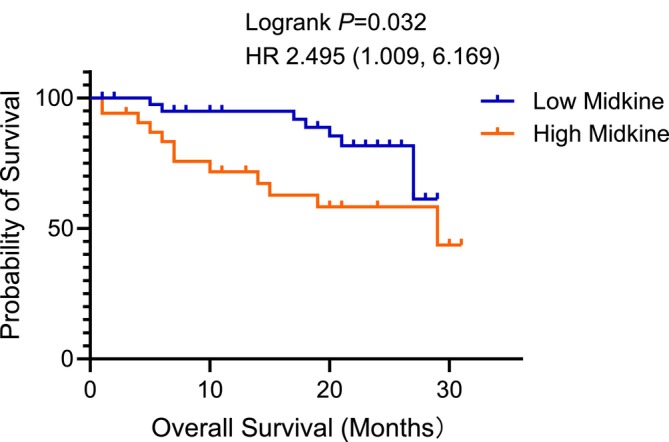
Efficacy of Midkine levels in predicting the overall survival for AFP negative HCC patients.

Results showed that in AFP negative HCC patients, high serum Midkine levels correlated with shorter survival when compared to low serum Midkine levels (*p* = 0.032), as indicated in Figure 2.

### 3.6 Midkine Levels as a Parameter for Treatment Response Evaluation in Comparison With Computed Tomography (CT) Scan and Magnetic Resonance Imaging (MRI) Practice

**FIGURE 3:**
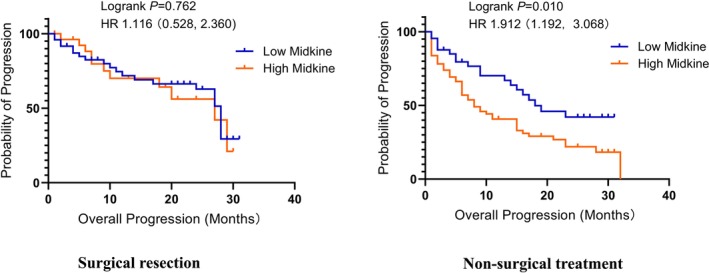
Efficacy of Midkine levels in predicting HCC progression after surgical resection and non‐surgical treatment.

Table 6 shows Midkine levels as treatment efficacy evaluation parameters. Briefly after 3 months of treatment, serum Mindkine and AFP were compared with CT scan or MRI examination to determine the usefulness of Midkine and AFP as treatment efficacy evaluation parameters. Patients were grouped into two groups: Group 1, tumor shrunk or stable with no changes. Group 2, tumor enlarged, new tumor loci appeared, positive lymph node metastasis, or portal vein thrombosis formation. Results showed that there was no significant difference between Midkine and CT or MRI of tumor size (*p* = 0.824 and 0.607, respectively). In 31 patients with significant tumor shrinkage or stable disease, 22 showed Midkine levels that were decreased, stable, or elevated > 15%. Nine patients showed Midkine levels which were increased ≥ 15%. Twenty‐five patients showed that AFP decreased, while six patients showed that AFP increased ≥ 15%. Figure 3 shows the efficacy of Midkine levels in predicting HCC progression after surgical resection and non‐surgical treatment. Results indicated that there was no significant difference in disease progression time between Midkine high and low levels in surgical resection treatment patients (*p* = 0.762); however, there was a significant difference in disease progression time between Midkine high and low levels in non‐surgical resection treatment patients (*p* = 0.010), and high Midkine levels had shorter disease progression time.

**TABLE 6:** Midkine levels as a parameter for treatment response evaluation and the comparison with computed tomography (CT) scan and magnetic resonance imaging (MRI).

		Total	Midkine			AFP		
			Decreased, no change, or elevated > 15%	Elevated ≥ 15%	*p*	Decreased, no change, or elevated > 15%	Elevated ≥ 15%	*p*
CT/MRI evaluation	Tumor shrunk or stable	31	22	9	0.824	25	6	0.607
	Progressive	15	11	4		9	6	
	Total	46	33	13		34	12	

*Note:* Includes all consecutive HCC patients (both AFP‐positive and AFP‐negative).

## 4 Discussion

Our results showed that HCC patients had the highest Midkine levels, followed by hepatic cholangiocarcinoma, chronic hepatitis, and liver cirrhosis patients; benign liver tumor patients displayed the lowest Midkine levels amongst all patient groups observed. These results indicate that Midkine is a biomarker for multiple clinical conditions; however, its high concentration in HCC makes it potentially clinically useful. As reported by Zhu et al. in 2013, Midkine levels were significantly elevated in HCC tissues and serum with a higher sensitivity than that of AFP [36]. Another study analyzed the performance of Midkine, dickkopf‐1, osteopontin, and AFP in HCC diagnosis, and compared them with liver cirrhosis, chronic liver disease, and healthy controls. They found that serum Midkine levels were higher in HCC patients when compared to liver cirrhosis, chronic liver disease, and healthy individuals. More than half of HCC patients were AFP negative. In the AFP‐negative HCC patients, 59.18% had elevated Midkine, and the calculated optimal cut‐off value was 0.44 ng/mL. Combinatory analysis of AFP (cut‐off value at ≥ 20 IU/mL) and Midkine (cut‐off value at ≥ 0.44 ng/mL) increased the diagnostic power to 76.7% of HCC cases [44].

Serum Midkine levels were associated with tumor size, ascites, high Child‐Pugh scores, high BCLC stages, and advanced clinical stages. There was no significant correlation between Midkine levels and liver cirrhosis or portal vein thrombosis. Zheng et al. found in an earlier study that Midkine was sensitive in diagnosing early‐stage HCC (BCLC‐A) and had the ability to diagnose AFP negative HCC patients, but they did not observe the relationship between Midkine and ascites and Child‐Pugh scores [38]. Our results also showed that Midkinie level was higher in advanced stages of HCC compared to early stages, indicating a potential correlation with tumor stage. Okada et al. found that serum Midkine positive patients had a greater number of tumor loci (≥ 2) [51].

Results from univariate and multivariate regression analysis showed that male sex, age, Midkine, PIVKA‐II, HBsAg (+), RBC count, peripheral blood monocyte percentage, and GGT to ALP ratio were significant indicators in HCC risk prediction. A previous study also suggested that Midkine was an independent predictive factor for HCC [53].

Results showed that in AFP negative HCC patients, high serum Midkine levels had shorter survival compared to low serum Midkine levels. We believe that this is a novel finding regarding the role of Midkine in HCC management.

Analysis of Midkine levels as a treatment efficacy and disease progression evaluation parameter indicated that there was no significant difference in disease progression time between Midkine high and low levels in surgical resection treatment patients; however, there was a significant difference in disease progression time between Midkine high and low levels in non‐surgical resection patients. High Midkine levels had shorter disease progression time, which means that serum Midkine is more useful in non‐surgical resection treatment patients than in surgical resection patients, as the former group tends to be of older age, advanced stage, and complicated clinical manifestations, while the latter group tends to have smaller tumor size and early clinical stage. There was no significant difference between serum Midkine levels and liver CT or MRI in treatment response evaluation, which means that the efficacy of serum Midkine levels in treatment response evaluation is similar to that of CT and MRI, and serum biomarker would have many advantages compared to CT and MRI, such as ease of access, low cost, and minimal invasiveness. Previous studies indicated that Midkine levels were significantly reduced after interventional treatment of the tumor. Thus, Midkine could be a promising indicator for treatment response as well as a predictor of recurrence [36, 38]. However, our evaluation methods were more specific and detailed.

## 5 Conclusions

Serum Midkine could be used as an independent predictive factor and as a marker for treatment response of HCC. Further studies on the value of serum Midkine levels in HCC recurrence prediction, follow‐up monitoring, and more rigorous studies on the association with survival should be performed.

Description of Supporting Information (Table S1): To evaluate the value of markers in predicting HCC in AFP negative patients by using the univariate and multivariate regression analysis, the results of univariate regression analysis indicated that 20 out of 62 clinical and laboratory parameters (Table S1) were significant (see Table 4), which were then further analyzed using multivariate regression analysis. Subsequently, eight parameters were found to be significant (see Table 5).

## 6 Limitations of This Study

Limitations in this study included: the patient sample size may not have been large enough; we were unable to include other etiologies related to HCC; our study was a single center study, and a multicentric study is needed in the future; we were unable to correlate the serum Midkine with the histological type of HCC.

## Author Contributions

Liejun Jiang designed the project, performed data statistical analysis, and managed project study; Yanfang Luo collected data and patient information; Qiuwei Lu and Jing Huang performed experiments.

## Funding

This study was funded by Guangxi Health Commission, No. S2022007.

## Ethics Statement

This study adhered to the Declaration of Helsinki for study involving humans or human data. The Ethic Committee/Institutional Review Board of the People's Hospital of Guangxi Zhuang Autonomous Region approved this study (No. KY‐SY‐2002‐007). Patients' informed consent was waived because the study only used patients' serum left over from routine testing, and patients' identifications were not disclosed. This was also approved by the Ethic Committee/Institutional Review Board of the People's Hospital of Guangxi Zhuang Autonomous Region.

## Consent

The authors have nothing to report.

## Conflicts of Interest

The authors thank Mindray Bio‐Medical Electronics Co. Ltd. for providing the Midkine ELISA reagent in this study.

## Supporting information


**Table S1:** jcla70255‐sup‐0001‐TableS1.docx.

## Data Availability

All data used in this study is available upon reasonable request and will be provided by the corresponding author.
