# Supplementary material for: Serum Midkine as a Biomarker for Hepatocellular Carcinoma Treatment Response and Prognostication
Source: J Clin Lab Anal. 2026 May 25;40(13):e70255. doi: 10.1002/jcla.70255 (PMC13371282; doi:10.1002/jcla.70255)
Supplement: Supplementary file 1 — Table S1: jcla70255‐sup‐0001‐TableS1.docx. [file JCLA-40-e70255-s001.docx]

**Table S1 Comparison of laboratory results and alcohol consumption between AFP negative HCC patients and non-cancerous subjects (hepatitis B, liver cirrhosis, benign liver tumors, and healthy controls)**

| Factors | Groups | n | Mean Rank | Sum of Ranks | Z | P |
| --- | --- | --- | --- | --- | --- | --- |
| Age | Controls | 370 | 211.74 | 78345 | -6.38 | 0.000 |
|  | AFP-NHCC | 92 | 310.96 | 28608 |  |  |
|  | Total | 462 |  |  |  |  |
| Sex | Controls | 371 | 242.61 | 90007 | -4.184 | 0.000 |
|  | AFP-NHCC | 92 | 189.23 | 17409 |  |  |
|  | Total | 463 |  |  |  |  |
| Alcohol consumption | Controls | 214 | 152.4 | 32614 | -0.438 | 0.661 |
|  | AFP-NHCC | 88 | 149.31 | 13139 |  |  |
|  | Total | 302 |  |  |  |  |
| Midkine | Controls | 371 | 204.08 | 75713.5 | -9.017 | 0.000 |
|  | AFP-NHCC | 92 | 344.59 | 31702.5 |  |  |
|  | Total | 463 |  |  |  |  |
| HBV-DNA | Controls | 138 | 94.85 | 13089 | -2.743 | 0.006 |
|  | AFP-NHCC | 65 | 117.18 | 7617 |  |  |
|  | Total | 203 |  |  |  |  |
| PIVKA-II | Controls | 252 | 140.68 | 35452 | -9.556 | 0.000 |
|  | AFP-NHCC | 89 | 256.84 | 22859 |  |  |
|  | Total | 341 |  |  |  |  |
| AFP-L3 | Controls | 284 | 175.82 | 49933.5 | -5.583 | 0.000 |
|  | AFP-NHCC | 83 | 211.98 | 17594.5 |  |  |
|  | Total | 367 |  |  |  |  |
| AFP-L3% | Controls | 283 | 176 | 49809 | -3.668 | 0.000 |
|  | AFP-NHCC | 83 | 209.06 | 17352 |  |  |
|  | Total | 366 |  |  |  |  |
| CA199 | Controls | 202 | 130.73 | 26406.5 | -1.566 | 0.117 |
|  | AFP-NHCC | 67 | 147.89 | 9908.5 |  |  |
|  | Total | 269 |  |  |  |  |
| CEA | Controls | 218 | 132.37 | 28857.5 | -4.074 | 0.000 |
|  | AFP-NHCC | 68 | 179.17 | 12183.5 |  |  |
|  | Total | 286 |  |  |  |  |
| HBsAg | Controls | 318 | 192.17 | 61110 | -4.772 | 0.000 |
|  | AFP-NHCC | 91 | 249.84 | 22735 |  |  |
|  | Total | 409 |  |  |  |  |
| HBeAg | Controls | 297 | 195.57 | 58084.5 | -0.814 | 0.416 |
|  | AFP-NHCC | 91 | 191.01 | 17381.5 |  |  |
|  | Total | 388 |  |  |  |  |
| HCVAb | Controls | 149 | 115.33 | 17184.5 | -1.323 | 0.186 |
|  | AFP-NHCC | 78 | 111.46 | 8693.5 |  |  |
|  | Total | 227 |  |  |  |  |
| TPAb | Controls | 163 | 125.54 | 20463 | -0.063 | 0.950 |
|  | AFP-NHCC | 87 | 125.43 | 10912 |  |  |
|  | Total | 250 |  |  |  |  |
| HIV | Controls | 167 | 127.02 | 21212.5 | -0.034 | 0.973 |
|  | AFP-NHCC | 86 | 126.96 | 10918.5 |  |  |
|  | Total | 253 |  |  |  |  |
| CRP | Controls | 166 | 118.64 | 19694 | -1.972 | 0.049 |
|  | AFP-NHCC | 83 | 137.72 | 11431 |  |  |
|  | Total | 249 |  |  |  |  |
| hsCRP | Controls | 88 | 60.18 | 5296 | -0.898 | 0.369 |
|  | AFP-NHCC | 35 | 66.57 | 2330 |  |  |
|  | Total | 123 |  |  |  |  |
| WBC | Controls | 354 | 225.56 | 79847 | -0.827 | 0.408 |
|  | AFP-NHCC | 91 | 213.05 | 19388 |  |  |
|  | Total | 445 |  |  |  |  |
| RBC | Controls | 354 | 231.06 | 81794 | -2.607 | 0.009 |
|  | AFP-NHCC | 91 | 191.66 | 17441 |  |  |
|  | Total | 445 |  |  |  |  |
| HBG | Controls | 354 | 231.68 | 82015.5 | -2.809 | 0.005 |
|  | AFP-NHCC | 91 | 189.23 | 17219.5 |  |  |
|  | Total | 445 |  |  |  |  |
| HCT | Controls | 354 | 232.37 | 82259.5 | -3.032 | 0.002 |
|  | AFP-NHCC | 91 | 186.54 | 16975.5 |  |  |
|  | Total | 445 |  |  |  |  |
| MCV | Controls | 354 | 222.28 | 78686.5 | -0.234 | 0.815 |
|  | AFP-NHCC | 91 | 225.81 | 20548.5 |  |  |
|  | Total | 445 |  |  |  |  |
| MCH | Controls | 354 | 221.77 | 78507.5 | -0.397 | 0.691 |
|  | AFP-NHCC | 91 | 227.77 | 20727.5 |  |  |
|  | Total | 445 |  |  |  |  |
| MCHC | Controls | 354 | 224.29 | 79398 | -0.417 | 0.677 |
|  | AFP-NHCC | 91 | 217.99 | 19837 |  |  |
|  | Total | 445 |  |  |  |  |
| PLT | Controls | 354 | 234.46 | 83000 | -3.709 | 0.000 |
|  | AFP-NHCC | 91 | 178.41 | 16235 |  |  |
|  | Total | 445 |  |  |  |  |
| Lymphocyte | Controls | 354 | 234.68 | 83077 | -3.779 | 0.000 |
|  | AFP-NHCC | 91 | 177.56 | 16158 |  |  |
|  | Total | 445 |  |  |  |  |
| Neutrophil | Controls | 354 | 218.19 | 77239 | -1.556 | 0.120 |
|  | AFP-NHCC | 91 | 241.71 | 21996 |  |  |
|  | Total | 445 |  |  |  |  |
| Monocyte | Controls | 354 | 205.53 | 72757.5 | -5.653 | 0.000 |
|  | AFP-NHCC | 91 | 290.96 | 26477.5 |  |  |
|  | Total | 445 |  |  |  |  |
| RDW-CV | Controls | 354 | 218.02 | 77180 | -1.611 | 0.107 |
|  | AFP-NHCC | 91 | 242.36 | 22055 |  |  |
|  | Total | 445 |  |  |  |  |
| RDW-SD | Controls | 353 | 218.24 | 77039 | -1.378 | 0.168 |
|  | AFP-NHCC | 91 | 239.02 | 21751 |  |  |
|  | Total | 444 |  |  |  |  |
| PDW | Controls | 328 | 211.97 | 69527.5 | -1.844 | 0.065 |
|  | AFP-NHCC | 84 | 185.13 | 15550.5 |  |  |
|  | Total | 412 |  |  |  |  |
| MPV | Controls | 328 | 197.32 | 64721.5 | -3.093 | 0.002 |
|  | AFP-NHCC | 84 | 242.34 | 20356.5 |  |  |
|  | Total | 412 |  |  |  |  |
| PCT | Controls | 329 | 213.5 | 70242.5 | -2.326 | 0.020 |
|  | AFP-NHCC | 84 | 181.53 | 15248.5 |  |  |
|  | Total | 413 |  |  |  |  |
| PT | Controls | 200 | 140.73 | 28145.5 | -1.445 | 0.148 |
|  | AFP-NHCC | 90 | 156.11 | 14049.5 |  |  |
|  | Total | 290 |  |  |  |  |
| PT-INR | Controls | 199 | 140.46 | 27951 | -1.375 | 0.169 |
|  | AFP-NHCC | 90 | 155.04 | 13954 |  |  |
|  | Total | 289 |  |  |  |  |
| FIB | Controls | 191 | 133.53 | 25503.5 | -2.246 | 0.025 |
|  | AFP-NHCC | 90 | 156.86 | 14117.5 |  |  |
|  | Total | 281 |  |  |  |  |
| TT | Controls | 190 | 135.47 | 25738.5 | -1.556 | 0.120 |
|  | AFP-NHCC | 90 | 151.13 | 13601.5 |  |  |
|  | Total | 280 |  |  |  |  |
| aPTT | Controls | 198 | 146.01 | 28909.5 | -0.457 | 0.648 |
|  | AFP-NHCC | 90 | 141.18 | 12706.5 |  |  |
|  | Total | 288 |  |  |  |  |
| ALT | Controls | 362 | 214.07 | 77492.5 | -4.329 | 0.000 |
|  | AFP-NHCC | 92 | 280.35 | 25792.5 |  |  |
|  | Total | 454 |  |  |  |  |
| AST | Controls | 362 | 209.97 | 76010 | -5.649 | 0.000 |
|  | AFP-NHCC | 92 | 296.47 | 27275 |  |  |
|  | Total | 454 |  |  |  |  |
| TP | Controls | 350 | 234.53 | 82086.5 | -4.184 | 0.000 |
|  | AFP-NHCC | 92 | 171.92 | 15816.5 |  |  |
|  | Total | 442 |  |  |  |  |
| ALB | Controls | 350 | 241.38 | 84482.5 | -6.382 | 0.000 |
|  | AFP-NHCC | 92 | 145.88 | 13420.5 |  |  |
|  | Total | 442 |  |  |  |  |
| GLB | Controls | 350 | 215.97 | 75589.5 | -1.775 | 0.076 |
|  | AFP-NHCC | 92 | 242.54 | 22313.5 |  |  |
|  | Total | 442 |  |  |  |  |
| A/G | Controls | 350 | 237.89 | 83263 | -5.263 | 0.000 |
|  | AFP-NHCC | 92 | 159.13 | 14640 |  |  |
|  | Total | 442 |  |  |  |  |
| TBIL | Controls | 359 | 219.03 | 78633.5 | -2.242 | 0.025 |
|  | AFP-NHCC | 92 | 253.18 | 23292.5 |  |  |
|  | Total | 451 |  |  |  |  |
| DBIL | Controls | 359 | 217.69 | 78151.5 | -2.675 | 0.007 |
|  | AFP-NHCC | 92 | 258.42 | 23774.5 |  |  |
|  | Total | 451 |  |  |  |  |
| IBIL | Controls | 360 | 222.33 | 80037 | -1.344 | 0.179 |
|  | AFP-NHCC | 92 | 242.84 | 22341 |  |  |
|  | Total | 452 |  |  |  |  |
| CHE | Controls | 297 | 206.73 | 61400 | -4.068 | 0.000 |
|  | AFP-NHCC | 90 | 151.98 | 13678 |  |  |
|  | Total | 387 |  |  |  |  |
| MAO | Controls | 297 | 198.16 | 58854.5 | -1.33 | 0.183 |
|  | AFP-NHCC | 90 | 180.26 | 16223.5 |  |  |
|  | Total | 387 |  |  |  |  |
| AFU | Controls | 296 | 183.26 | 54245 | -3.27 | 0.001 |
|  | AFP-NHCC | 90 | 227.18 | 20446 |  |  |
|  | Total | 386 |  |  |  |  |
| ADA | Controls | 296 | 171.98 | 50907.5 | -6.871 | 0.000 |
|  | AFP-NHCC | 90 | 264.26 | 23783.5 |  |  |
|  | Total | 386 |  |  |  |  |
| ALP | Controls | 336 | 203.16 | 68261 | -3.626 | 0.000 |
|  | AFP-NHCC | 92 | 255.92 | 23545 |  |  |
|  | Total | 428 |  |  |  |  |
| TBA | Controls | 298 | 185.59 | 55307 | -2.846 | 0.004 |
|  | AFP-NHCC | 90 | 223.99 | 20159 |  |  |
|  | Total | 388 |  |  |  |  |
| GGT | Controls | 360 | 208.42 | 75032.5 | -5.821 | 0.000 |
|  | AFP-NHCC | 92 | 297.23 | 27345.5 |  |  |
|  | Total | 452 |  |  |  |  |
| AST/ALT | Controls | 362 | 225.19 | 81519 | -0.744 | 0.457 |
|  | AFP-NHCC | 92 | 236.59 | 21766 |  |  |
|  | Total | 454 |  |  |  |  |
| GGT/AST | Controls | 362 | 218.83 | 79218 | -2.792 | 0.005 |
|  | AFP-NHCC | 92 | 261.6 | 24067 |  |  |
|  | Total | 454 |  |  |  |  |
| GGT/ALP | Controls | 336 | 198.66 | 66749.5 | -5.064 | 0.000 |
|  | AFP-NHCC | 92 | 272.35 | 25056.5 |  |  |
|  | Total | 428 |  |  |  |  |
| PA | Controls | 285 | 198.65 | 56616 | -3.57 | 0.000 |
|  | AFP-NHCC | 89 | 151.79 | 13509 |  |  |
|  | Total | 374 |  |  |  |  |
| Urea | Controls | 324 | 201.08 | 65148.5 | -2.073 | 0.038 |
|  | AFP-NHCC | 90 | 230.63 | 20756.5 |  |  |
|  | Total | 414 |  |  |  |  |
| CREA | Controls | 324 | 200.68 | 65021 | -2.2 | 0.028 |
|  | AFP-NHCC | 90 | 232.04 | 20884 |  |  |
|  | Total | 414 |  |  |  |  |
| UA | Controls | 325 | 209.05 | 67942.5 | -0.34 | 0.734 |
|  | AFP-NHCC | 90 | 204.19 | 18377.5 |  |  |
|  | Total | 415 |  |  |  |  |
| HCO3 | Controls | 314 | 207.83 | 65260 | -1.715 | 0.086 |
|  | AFP-NHCC | 90 | 183.89 | 16550 |  |  |
|  | Total | 404 |  |  |  |  |
